# Metabolic Reliance on Photosynthesis Depends on Both Irradiance and Prey Availability in the Mixotrophic Ciliate, *Strombidium* cf. *basimorphum*

**DOI:** 10.3389/fmicb.2021.642600

**Published:** 2021-06-17

**Authors:** Erin Ann Hughes, Maira Maselli, Helle Sørensen, Per Juel Hansen

**Affiliations:** ^1^Marine Biological Section, Biological Institute, University of Copenhagen, Copenhagen, Denmark; ^2^Data Science Lab, Department of Mathematical Sciences, University of Copenhagen, Copenhagen, Denmark

**Keywords:** ciliates, photosynthesis, ingestion, GNCM, *Strombidium*, irradiance, mixotrophy

## Abstract

Many species of the ciliate genus *Strombidium* can acquire functional chloroplasts from a wide range of algal prey and are thus classified as generalist non-constitutive mixotrophs. Little, however, is known about the influence of irradiance and prey availability on their ability to exploit the photosynthetic potential of the chloroplasts, and how this may explain their spatial and temporal distribution in nature. In this study, inorganic carbon uptake, growth, and ingestion rates were measured for *S.* cf. *basimorphum* under three different irradiances (10, 40, and 120 μmol photons m^–2^ s^–1^) when acclimated to three different prey densities (5 × 10^3^, 1 × 10^4^, and 4 × 10^4^ cells mL^–1^), as well as when allowed to deplete the prey. After prey depletion, cultures survived without prey longest (∼6 days) at the medium irradiance treatment (40 μmol photons m^–2^ s^–1^), while ciliate density, inorganic carbon uptake rates, and cellular chl-*a* content declined fastest at the highest irradiance treatment. This indicates that the ciliates may be unable to maintain the chloroplasts functionally without replacement at high irradiances. Ingestion rates were not shown to be significantly influenced by irradiance. The maximum gross growth efficiency (GGE) in this study (1.1) was measured in cultures exposed to the medium test irradiance and lowest prey density treatment (5 × 10^3^ cells mL^–1^). The relative contribution of inorganic carbon uptake to the ciliate carbon budget was also highest in this treatment (42%). A secondary GGE peak (0.99) occurred when cultures were exposed to the highest test irradiance and the medium prey density. These and other results suggest that *S.* cf. *basimorphum*, and other generalist non-constitutive mixotrophs, can flexibly exploit many different environmental conditions across the globe.

## Introduction

Mixotrophy in protists is generically defined as a nutritional strategy that combines phagotrophy and phototrophy (Flynn et al., 2019). An increasing number of protist species have been shown to utilize varying degrees of mixotrophy, and thus the perceived prevalence and relevance of this nutritional strategy to the marine food web and nutrient cycles is also rising. Subsequently, more ecophysiological studies of mixotrophic organisms have been conducted in recent years and researchers have been able to incorporate mixotrophic functions into ecosystem models of aquatic environments. Already, the ability of mixotrophic organisms to utilize multiple avenues of energy and nutrient acquisition has been used to predict an increased efficiency in the transfer of biomass through trophic levels (Ward and Follows, 2016).

Mixotrophy in protists may be organized in several different ways. Mitra et al. (2016) categorized mixotrophic strategies into two primary groups: constitutive mixotrophs that have their own innate photosystems and non-constitutive mixotrophs that must acquire or utilize photosystems taken from prey. Non-constitutive mixotrophs can then further be split into two subcategories: generalist non-constitutive mixotrophs (GNCMs) that can acquire the ability to fixate carbon from multiple prey sources and specialist non-constitutive mixotrophs (SNCMs) that can only utilize specific species or genera of prey. While different strategies along the spectrum of mixotrophy may be more prevalent at different latitudes or times of year (Stoecker et al., 2009; Leles et al., 2017), mixotrophy as a whole is exceedingly common and fundamental to many aquatic environments (Sanders, 1991; Mitra et al., 2014; Stoecker and Lavrentyev, 2018).

Non-constitutive mixotrophy is a particularly dominant nutritional strategy within the ubiquitous planktonic phylum, Ciliophora (Stoecker et al., 2009). While ciliates do not have the innate ability to photosynthesize, some can sequester and utilize sequestered chloroplasts from their prey, a mechanism known as kleptoplasty. In marine waters, the most well-studied NCM ciliates are the bloom-forming red *Mesodinium* spp. These species are considered SNCMs due to the close association between hosts and their sequestered prey organelles. Consequently, red *Mesodinium* spp. acquire a vast majority of carbon from photosynthesis (as opposed to prey ingestion) (Hansen et al., 2013) and often exhibit the ability to survive for at least 1 month without food (Johnson and Stoecker, 2005; Smith and Hansen, 2007).

Another pervasive ciliate subclass is Oligotrichia, which contains both heterotrophic and mixotrophic species. Unlike the red *Mesodinium* spp., the mixotrophic oligotrichs have been shown to retain and utilize chloroplasts from many different species of algal prey (Johnson and Beaudoin, 2019; Maselli et al., 2020), and thus, are generally considered GNCMs. Mixotrophic oligotrichs are believed to depend on frequent reacquisition of prey plastids, as they do not appear to express genes related to plastid maintenance and replication (Santoferrara et al., 2014; Mcmanus et al., 2018). These plastidic oligotrichs make up, on average, 30% of ciliate biomass (Dolan and Pérez, 2000; Stoecker et al., 2009), and during spring- and summer-time peaks, they can even comprise >90% of ciliate biomass (Bernard and Rassoulzadegan, 1994; Haraguchi et al., 2018). Additionally, plastidic ciliates can represent anywhere from 4 to over 50% of total chlorophyll-a (chl-*a*) in a system, making them of vital importance for overall primary production (Putt, 1990a; Stoecker et al., 2014; Franzè and Lavrentyev, 2017). Thus, the prevalence of GNCM ciliates within natural ecosystems makes it exceedingly important to better quantify their physiological processes.

To date, most studies on oligotrich ciliates have focused on the effects of prey depletion and subsequent starvation on ingestion, photosynthesis, and growth responses, sometimes comparing responses in the light and in the dark (e.g., Stoecker and Michaels, 1991; Montagnes, 1996). However, as GNCMs likely gain a competitive advantage in nature via their nutritional flexibility, it is important to further elucidate the specific interactions between prey availability and GNCM inorganic carbon uptake rate – a topic largely ignored in current literature. Photosynthesis in GNCM ciliates may be strictly beholden to the number of prey chloroplasts that are available and, therefore, would exhibit a clear positive correlation with prey concentration. However, if the ciliates do have some ability to regulate the amount of photosynthesis performed by the stolen plastids, they may upregulate photosynthetic activity in response to reduced access to organic carbon from prey and downregulate photosynthesis when they are exposed to environments with high prey density.

Additionally, very few papers have explored how the physiological responses of GNCMs may change under different light conditions. Indeed, in one of the only studies to quantify a mixotrophic oligotrich’s responses to light, Stoecker et al. (1988b) showed that unlike the light-dependent grazing seen in many SNCMs (Moeller et al., 2019), *Laboea strobila*’s ingestion rate appeared to be independent of light. Additionally, *L. strobila*, as well as several *Strombidium* species, were shown to exhibit an inorganic carbon uptake rate that increased with increasing irradiance up to a point of saturation (Stoecker et al.,1988a,b). Other than these two studies, little is known about the effect of irradiance on ingestion, growth, photosynthesis, and response to prey depletion in GNCMs. Quantifying this behavior is crucial to the development of a deeper understanding of how mixotrophic strategies interact with the environment and affect the planktonic community structure.

To this end, this study aims to gain an understanding of mixotrophy in *Strombidium* cf. *basimorphum*. *S.* cf. *basimorphum* is a ubiquitous species that can be found across the globe (Martin and Montagnes, 1993; Liu et al., 2011; Orsi et al., 2018). However, its mixotrophic capabilities have only recently been proven in isolates from Danish coastal water (Maselli et al., 2020). Cultures of the same isolate, identified based on 18S and 28S gene sequences (Maselli et al., 2020, GenBank accession number MT349841 and MT420874), have been used in this study to monitor how light affects: (1) photosynthesis and survival of *S.* cf. *basimorphum* when starved after prey depletion and (2) growth and photosynthesis of the ciliate at different prey densities. With this information, we can gain insight into which situations mixotrophy may lend an advantage to *S.* cf. *basimorphum* when compared to other planktonic species that employ different nutritional acquisition strategies.

## Materials and Methods

### Culture of Organisms

*Strombidium* cf. *basimorphum* cultures were obtained from the culture collection of the Marine Biological Section in Helsingør, Denmark. This species was originally collected and isolated in June 2018 from Roskilde Fjord, Denmark (Maselli et al., 2020). Ciliate cultures were maintained on the cryptophyte prey *Teleaulax amphioxeia*, which was provided by the Scandinavian Culture Collection of Algae and Protozoa (SCCAP, strain number: K-1837). All cultures were grown at a temperature of 15°C in filtered (Whatman, GF/F) and enriched f/20 seawater medium at a salinity of 15 ± 1. Light was provided by cool-white fluorescent lights at a 14:10 h light:dark cycle with an intensity of 10, 40, or 120 μmol photons m^–2^ s^–1^, depending on the experiment.

### Experiments

To get a comprehensive understanding of the effects of irradiance and prey availability on *S.* cf. *basimorphum*, two sets of experiments were designed. Both experiments utilized three light levels: 10, 40, and 120 μmol photons m^–2^ s^–1^; these will henceforth be referred to as I_10_, I_40_, and I_120_, respectively. These irradiances were selected to represent the range of light in which *S.* cf. *basimorphum* can typically be found. I_10_ represents an irradiance that could be found at the base of the pycnocline, where light levels can often be around 1% of surface irradiance. I_40_ represents irradiance levels potentially found at the top of the pycnocline, and I_120_ is the average that can be found within the upper mixed layers of the euphotic zone (Abdelrhman, 2016). Both experiments investigate changes in growth/mortality, ingestion, and inorganic carbon uptake rates, while also measuring ciliate cell biovolume and chl-*a* content. The first experiment explores differences in the physiologic responses of well-fed ciliate cells when they are subject to prey depletion at each of the three experimental light levels (the starvation experiment). The second investigates differences in the physiologic responses of ciliates acclimated to three different prey concentrations at each of the three light levels (the acclimation experiment).

#### Experiment 1: Starvation Experiment

For these experiments, effects of irradiance on photosynthetic and survival responses of *S.* cf. *basimorphum* when starved of prey were studied. The experiments were terminated after 13 days, or after the ciliate density was <5 cells mL^–1^. For a minimum of 5 days before the initiation of experiments for each light condition, 800 mL of *S.* cf. *basimorphum* mixed culture (contained in a 1L glass culture flask) were acclimatized to the experimental irradiance and fed light-acclimatized *T. amphioxeia* cells at prey concentrations that were replenished daily to 4 × 10^4^ cells mL^–1^. When the ciliate density reached approximately 175 cells mL^–1^, the experiment was initiated (day 0) by splitting the acclimated culture into three 200 mL triplicates that were contained in 500 mL glass culture flasks. Then, *T. amphioxeia* cells were added to the experimental cultures so that the algal density of the culture reached a saturating prey density of ∼3–3.5 × 10^4^ cells mL^–1^. Subsamples (5–10 mL) were collected, at minimum, on days 2, 5, 7, and 9, as well as every 2 days thereafter until the termination of each experiment. These subsamples were used to measure the densities (individuals mL^–1^), growth rates (cell divisions d^–1^), photosynthetic rates (pg C cell^–1^h^–1^), and chlorophyll content (pg chl*-a* cell^–1^) of both ciliates and algae, as well as to measure the ciliates’ cell volumes (μm^3^). Monocultures of *Teleaulax amphioxeia* were run in parallel with the mixed cultures until there were no measurable prey densities within ciliate cultures. Algal density and growth were measured in the monocultures before they were diluted with fresh f/20 media on each sampling day to match the algal densities found in the experimental cultures. The monocultures were also sampled for photosynthetic rate and chlorophyll content on the same days as the experimental cultures.

#### Experiment 2: Acclimation Experiment

**FIGURE 1:**
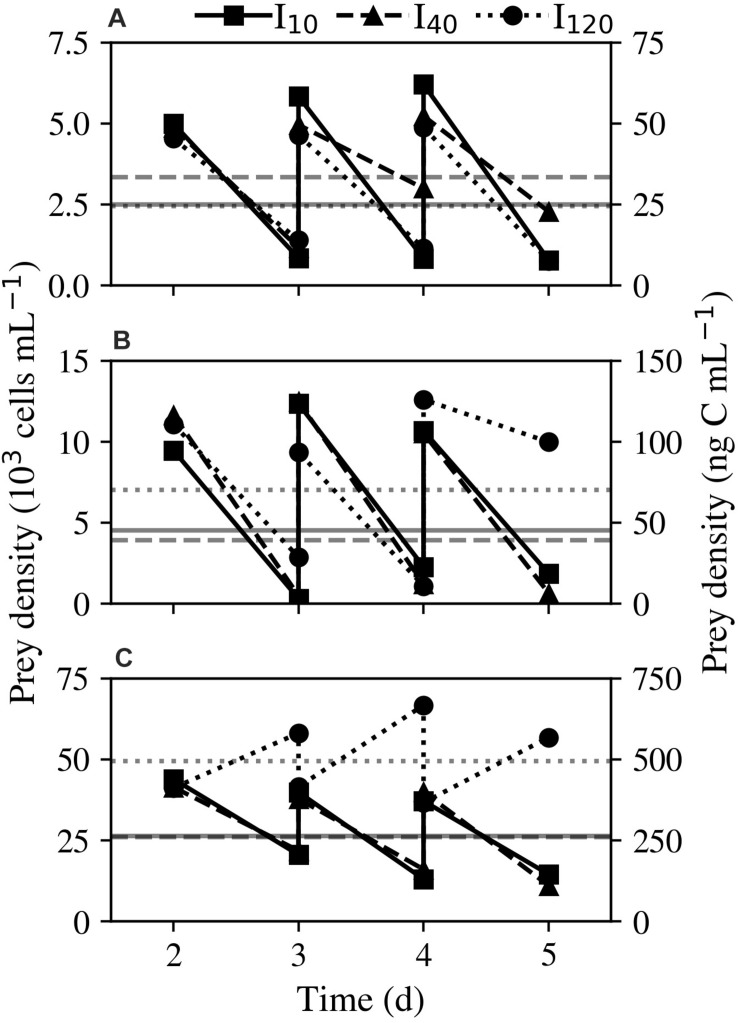
Experiment 2: Acclimation of *Strombidium* cf. *basimorphum* to different prey densities: **(A)** 50, **(B)** 100, and **(C)** 400 ng C mL^–1^. Dilutions were made daily to return cultures to the experimental prey levels. Solid, dashed, and dotted lines denote light treatments I_10_, I_40_, and I_120_, respectively. Horizontal gray lines represent the average prey density of each culture over all sampling days. Data points are means (*n* = 3).

Experiment 2 was carried out to study the effects of light and prey density on growth, ingestion, and photosynthetic rates of *Strombidium* cf. *basimorphum* using *Teleaulax amphioxeia* as prey. Cultures were acclimatized to each light condition for a minimum of 5 days prior to the initiation of all experiments. The duration of the experiment was 5 days, with days 1 and 2 serving to acclimatize cultures to the experimental prey densities. Monocultures of *T. amphioxeia* were run as controls alongside mixed cultures. The experiments were carried out at the same light levels as for the previous experiment: I_10_, I_40_, and I_120_. A preliminary experiment was carried out to provide a rough estimate of the minimum prey density required for culture survival (see Supplementary Material 1 for a detailed outline of this experiment). Then, for each light treatment, three different prey densities were tested: 5 × 10^3^, 1 × 10^4^, and 4 × 10^4^ cells mL^–1^. These prey densities were chosen to cover a range of ecologically relevant prey concentrations (Levinsen et al., 2000; Haraguchi et al., 2018), with the highest density treatment representing prey concentrations that can occur during spring phytoplankton blooms, and the two lower densities representing prey concentrations more typically found throughout the rest of the year. *S.* cf. *basimorphum* density was maintained at 15 individuals mL^–1^. Ciliate and prey densities were adjusted every day by dilution with fresh media and addition of algae from monocultures (Figure 1). During days 1–2, 800 mL of both the mixed cultures and control monocultures were kept in 1 L glass culture flasks. At the end of day 2, the acclimatized cultures were split into triplicates by placing 200 mL of each culture into 500 mL glass culture flasks. Subsamples (5–10 mL) were taken daily at a fixed time for all 5 days of the experiment to discern algal and ciliate densities. On days 3–5 of the experiment, additional subsamples were taken to measure cell volume, photosynthetic rate, and chlorophyll content as described previously.

### Techniques

#### Enumeration of Cells

For enumeration of ciliates, 2 mL samples were placed in 24-well tissue culture plates, fixed in Lugol’s solution (final concentration 1%), and subsequently counted on an inverted light microscope (Olympus CKX53) at 50× magnification. Generally, this was repeated up to six times, until a minimum of 200 individuals was counted. For the very dilute cultures found at the end of the starvation experiment, this procedure was repeated up to 12 times. Algal prey was counted using a CytoFLEX flow cytometer (Beckman Coulter, United States) based on fluorescence patterns and cell size from side angle light scatter (Olson et al., 1991). Algal densities were then converted to their C (carbon) equivalents by assuming that each cell contains 10 pg C, based on average cell volume (Menden-Deuer and Lessard, 2000), as in Maselli et al. (2020).

#### Ciliate Cell Volume

The length and width of the ciliate cells preserved with Lugol’s solution were measured using an Olympus TH4-200 light microscope, which was set to a magnification of 200x and connected to an Olympus DP73 camera. Measurements were taken utilizing the CellSense software. *S.* cf. *basimorphum* cells were determined to be either spherical or ellipsoidal, and biovolume (in μm^3^) was calculated accordingly.

#### Growth and Ingestion Rates

For both acclimation and starvation experiments, the daily growth rate of *Strombidium* cf. *basimorphum* (*μ_*y*_*) and prey cells (*μ_*x*_*) were calculated assuming exponential growth (Eq. 1):

(1) $$\mu =\frac{{\text{lnN}}_{1}-{\text{lnN}}_{0}}{\text{t}}$$

where N_0_ and N_1_ refer to the number of cells mL^–1^ at the start (t_0_) and end (t_1_) of each incubation period, respectively, and t is the time interval between samplings (in days unless otherwise noted).

The average algal and ciliate densities in cultures were used to calculate the culture’s clearance rate and, ultimately, the ingestion rate. This method was first suggested by Frost (1972) and later modified by Heinbokel (1978). Within this article, we calculated the amount of prey ingested throughout a 24 h period. The actual ingestion rate at any given time within each day may vary significantly based on the light:dark cycles and the specific amount of prey available. This is particularly important to note for cultures within the two lowest prey concentration treatments (5 × 10^3^ and 1 × 10^4^ cells mL^–1^), as the prey were drastically depleted to near-imperceptible levels within 24 h. Thus, the daily ingestion rates in this paper are reported as a function of our three initial prey density treatment levels, which should not be misunderstood as the average prey density over each day. These calculations of daily ingestion rate were also used for calculations of gross growth efficiency and carbon acquisition.

#### Chlorophyll-*a* Content

For ciliate chl-*a* measurements, twenty ciliate cells from each experimental bottle were picked with a drawn micropipette, rinsed twice in fresh f/20 media to remove prey cells, and added to 2 mL of 96% ethanol. Algal chl-*a* content was measured by collecting 5 mL of algal culture onto glass microfiber filters (Whatman, GF/F) before the filters were placed in 5 mL of 96% ethanol. All samples were then stored in the dark at 4°C for 24 h, after which chl-*a* was measured using a Turner Trilogy Fluorometer set with a chl-*a* (non-acid) insert.

#### Inorganic Carbon Uptake Rate

Inorganic carbon uptake rates were measured using the “single cell method” (Rivkin and Seliger, 1981). For each experimental ciliate culture, two 23 mL glass scintillation vials were prepared with 2 mL of fresh f/20 media. Twenty ciliate cells per culture bottle were then picked using a drawn Pasteur pipette and washed twice in fresh f/20 media to separate them from any remaining prey before being added to the scintillation vials. For experiments in which ciliates were given *T. amphioxeia* as prey, control monocultures of this algae were maintained alongside mixed cultures. For these control cultures, two scintillation vials containing 2 mL of monoculture were prepared from each control culture. Then, for both ciliate and control algal cultures, 20 μL of NaH^14^CO_3_^–^ stock solution (specific activity 100 μCi ml^–1^) was added to each vial; one vial for every culture was incubated in the experimental light conditions, while the second vial was wrapped in aluminum foil and incubated in the dark. The incubation lasted 3 h, whereafter subsamples of 100 μL from each of the incubated vials was added to a separate vial containing 200 μL phenylethylamine. These new vials were used to measure the specific activity of the medium. The remaining 1.9 mL was treated with 2 mL of 10% acetic acid in methanol to remove all the inorganic carbon. The vials were dried overnight at 64°C before the resulting residue was re-dissolved in 1.5 mL distilled water. 10 mL of Ultima Gold^TM^ XR scintillation fluid was added to all vials (including those for specific activity) and activity was measured using a Packard 1500 TriCarb liquid scintillation counter. Daily carbon incorporation rates, P (pg C cell^–1^ d^–1^) were calculated by multiplying the hourly rates (Eq. 2) by the duration of the daily light period (14 h):

(2) $$\text{P}=\frac{\left[\frac{{\text{D}}_{\text{l}}-{\text{D}}_{\text{d}}}{\text{N}}\right]\,{\text{C}}_{\text{m}}\times 14\times \,10^{6}}{\text{SA}\times t}$$

where D_*l*_ and D_*d*_ refer to the disintegration per minute in the light and dark, respectively. SA refers to specific activity (disintegrations per minute) of ^14^C in the medium. N is the number of cells measured, and C_*m*_ refers to the inorganic carbon content of the medium (μg C mL^–1^). t is the incubation time in hours. 10^6^ is used to convert μg to pg.

The total inorganic carbon of the culture medium was measured with a Shimadzu TOC-L analyzer using 25 mL media samples secured in screw-top glass vials so that no air was present in the sample.

#### Gross Growth Efficiency and Carbon Content

Gross growth efficiency (GGE, Eq. 6) was calculated as the percentage of the ingested carbon (pg C cell^–1^ d^–1^) effectively converted into new ciliate biomass (Eq. 5). *μ*_*yC*_ is reported in pg C cell^–1^ d^–1^ and is the C-specific growth rate. The calculation of ingested carbon (C_*I*_, Eq. 3) utilizes the aforementioned assumption that each ingested algal cell contains 10 pg C. Ciliate carbon content (C_*y*_, Eq. 4) was calculated by multiplying the previously calculated biovolume by a conversion factor of 0.19 pg C μm^–3^ (Putt and Stoecker, 1989).

(3) $${\text{C}}_{\text{I}}=\text{I}\times 10$$

(4) $${\text{C}}_{\text{y}}=0.19\times {\text{B}}_{\text{v}}$$

(5) $$\,\mu_{\text{yC}}=\mu_{\text{y}}\times C_{\text{y}}$$

(6) $$\text{GGE}=\frac{\mu_{\text{yC}}}{{\text{C}}_{\text{I}}}$$

#### Statistical Methods

As data from triplicates in Experiment 1 came from the same acclimatized culture, we did not do formal statistical testing and this data is used for trend description and visualization. As two independent variables were tested in Experiment 2, this data was able to undergo statistical testing. Data were analyzed with a linear mixed model (LMM) with fixed effects of irradiance, prey density, and day (categorical variables, three levels each) and random effects of the source bottle (nine levels) and sample bottle (27 levels). Some outcomes were log-transformed to obtain variance homogeneity. Notice that no interactions were included in the LMM due to the experimental design; thus, only main/overall effects were tested. These tests were carried out as *F*-tests (with Satterthwaite’s approximation for degrees of freedom, where necessary). Pairwise comparisons were carried out with Tukey-adjusted *P*-values, and letters were used to communicate statistically significant groups (at significance level 0.05). The thirty measurements of cell volume from each sample bottle did not correspond to specific days, so no fixed effect of Day was included for cell volume. The statistical software environment R version 3.6.3 (R Core Team, 2020) and packages lmerTest (Kuznetsova et al., 2017), emmeans (Lenth, 2020), and multcomp (Hothorn et al., 2008) were used for the analyses. All results are presented graphically as means ± STD and graphs were created using Python’s matplotlib library (Hunter, 2007).

## Results

### Experiment 1: Starvation Experiment

#### Growth and Mortality

**FIGURE 2:**
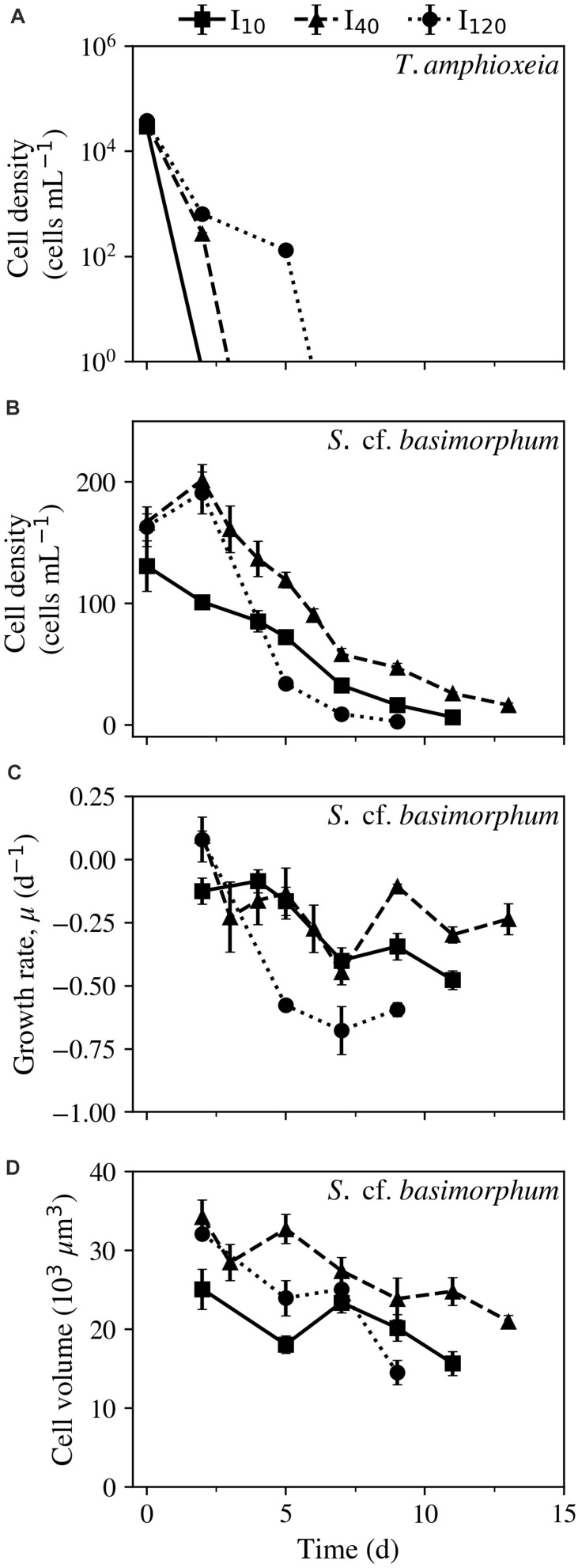
Experiment 1: Physiological responses of *Strombidium* cf. *basimorphum* to prey starvation at three different light treatments (I_10_, I_40_, and I_120_) as a function of time (d). **(A,B)** Cell densities of *T. amphioxeia* and *S.* cf. *basimorphum*, respectively. **(C)** Growth rate of *S.* cf. *basimorphum*. **(D)** Biovolume of Lugol-fixed ciliate cells. Solid, dashed, and dotted lines denote light treatments I_10_, I_40_, and I_120_, respectively. Data points are means ± STD (*n* = 3).

Ciliate cultures initially experienced growth while food was still available (between days 0 and 2) at light levels of 40 (I_40_) and 120 (I_120_) μmol photons m^–2^ s^–1^, after which the populations began to die out. In cultures kept at 10 (I_10_) μmol photons m^–2^ s^–1^, prey was completely depleted by day 2, at which point populations were already experiencing net mortality (Figures 2A,B). The steepest decline occurred in the I_120_ treatment, which reached an absolute maximum mortality rate on day 7 of −0.68 ± 0.1 d^–1^ (mean ± STD) (Figure 2C). By contrast, cultures experienced a much more gradual decline at I_10_ and I_40_, with maximum mortality rates of −0.48 ± 0.04 and −0.45 ± 0.05 d^–1^, respectively. Biovolumes of the cells at all irradiances were highest while ciliates were still well-fed (day 2), though already at this time point, ciliates at I_10_ were notably smaller than their counterparts at higher irradiances (Figure 2D). A steep decline in volume was observed between days 2 and 5 at irradiances of I_10_ and I_120_. At these light treatments, cells also experienced a relatively steep decline in cell biovolumes on the final day of the experiments. Cultures at I_40_ still saw a net decline in biovolume, but it occurred more gradually, and the cells were much larger at the termination of the experiment than in the other two treatments.

#### Chlorophyll-*a* and Photosynthesis

**FIGURE 3:**
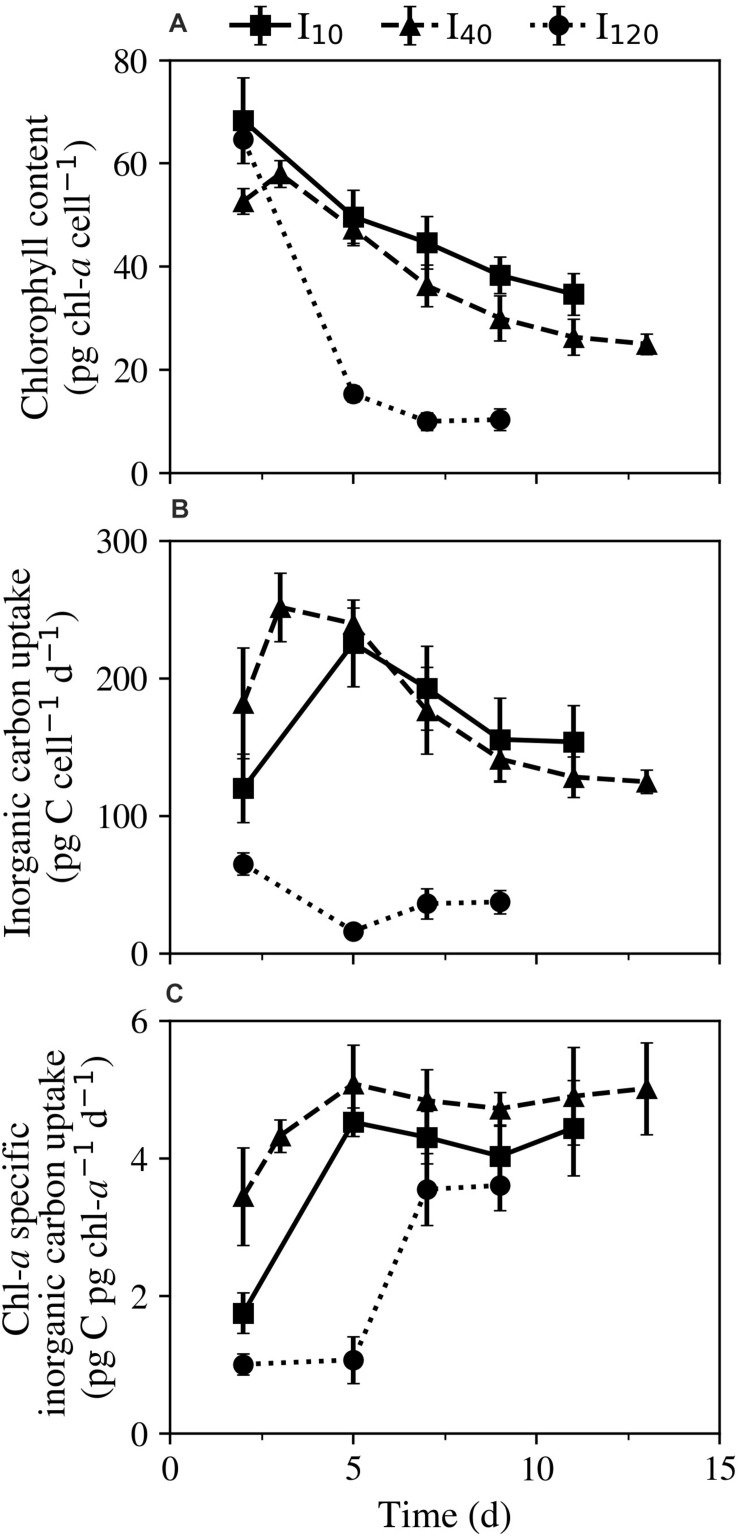
Experiment 1: Physiological responses of *Strombidium* cf. *basimorphum* to starvation at three different light treatments (I_10_, I_40_, and I_120_) as a function of time (d). **(A)** Cellular chl-*a* content. **(B)** Cellular photosynthetic rate. **(C)** Chl-*a* specific photosynthetic rate. Solid, dashed, and dotted lines denote light treatments I_10_, I_40_, and I_120_, respectively. Data points are means ± STD (n = 3).

Throughout the experiment, all treatments experienced a steep net decline in cellular chl-*a* content (Figure 3A). The ciliates’ ability to maintain their cellular chl-*a* after prey was depleted had an inverse relationship with irradiance. Following the trend exhibited in mortality rate, the fastest decline in chl-*a* occurred between days 2 and 5 at I_120_, where cultures went from 64.7 ± 4.6 pg chl-*a* cell^–1^ on day 2 to 15.3 ± 1.5 pg chl-*a* cell^–1^ on day 5. Cultures kept at I_10_ exhibited the greatest ability to maintain chl-*a* levels, starting on day 2 at 68.3 ± 8.3 pg chl-*a* cell^–1^, and ending on day 11 at 34.7 ± 4.0 pg chl-*a* cell^–1^.

At the two lower irradiances, a notable increase in cellular inorganic carbon incorporation between days 2 and 5 was observed, after which cellular inorganic carbon uptake rates declined until the cessation of the experiments on days 11 and 13, respectively. Across all days of the experiment, photosynthetic rates ranged from 120 to 226 pg C cell^–1^ day^–1^, and 125 to 252 pg C cell^–1^ day^–1^ at I_10_ and I_40_, respectively (Figure 3B). Photosynthetic rates at I_120_ were considerably lower than at the other two light treatments and ranged from 16.2 to 65.1 pg C cell^–1^ day^–1^. Chl-*a* specific inorganic carbon uptake rates (pg C pg chl-*a*^–1^ d^–1^) generally increased in all cultures over the course of the experiment. Cultures kept at I_10_ and I_40_ both experienced an increase in chl-*a* specific photosynthetic rate until day 5, thereafter seeing only minor fluctuations until the experiments were ended (Figure 3C).

### Experiment 2: Acclimation Experiment

#### Growth and Ingestion

**FIGURE 4:**
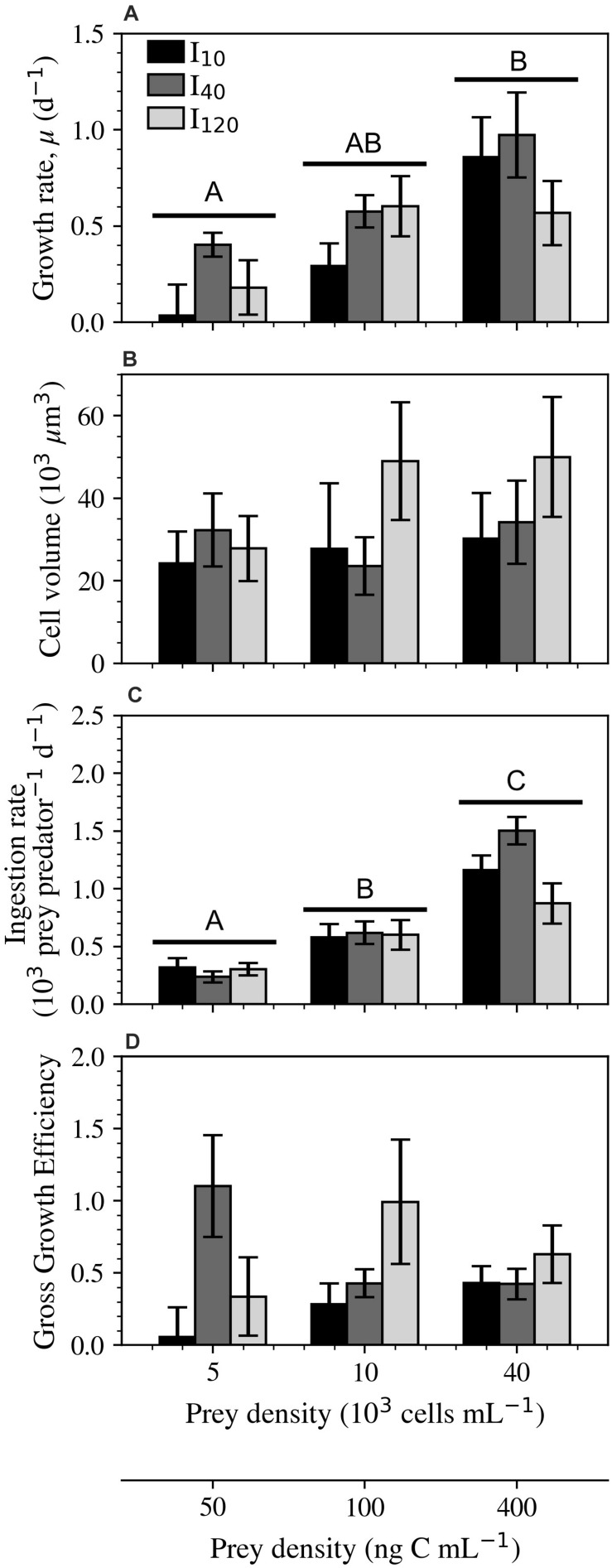
Experiment 2: Physiological responses of *Strombidium* cf. *basimorphum* at the three light (I_10_, I_40_, and I_120_) and three prey density treatments (50, 100, and 400 ng C mL^–1^). **(A)** Growth rate (*n* = 9). **(B)** Biovolume of Lugol-fixed ciliate cells (*n* = 90). **(C)** Ingestion rate (*n* = 9). **(D)** GGE (*n* = 9). Black, dark gray, and light gray bars denote light treatments I_10_, I_40_, and I_120_, respectively. Data points are means ± STD. Irradiance was not proven to be statistically significant for any of the displayed variables; the letters, therefore, represent statistically similar groupings based only on the overall effects of prey density across all irradiance treatments. Subplots without letters did not show significant effects of prey density or irradiance.

Growth rates of *S.* cf. *basimorphum* were significantly influenced by prey density, whereas the statistical significance of irradiance could not be proven in this experiment (Figure 4A). Cultures kept at the lowest irradiance (I_10_) saw the greatest change in growth rate across the range of prey densities. Cultures kept at I_40_ exhibited the highest average growth rates in the low prey density treatment. Ciliates at this intermediate irradiance also experienced some of the highest growth rates at the highest prey density, however, this comparison becomes less striking when biovolume is also considered (Supplementary Figure 2). Compared to cells subjected to other treatments, ciliates grown at the highest irradiance had nearly twice as much biovolume when supplied with a prey density at or above 100 ng C mL^–1^ (Figure 4B). See Supplementary Figure 3 for size comparisons of Lugol-fixed *S.* cf. *basimorphum* cells and Supplementary Table 3 for the average linear dimensions of cells in all nine treatments.

Only prey density was shown to have a significant effect on ingestion rates. In particular, ingestion rates exhibited a steep increase of 583, 884, and 271 prey predator^–1^ d^–1^ between 100 and 400 ng C mL^–1^ for I_10_, I_40_, and I_120_, respectively (Figure 4C). GGE was consistently lowest at I_10_, where a maximum efficiency of 0.43 ± 0.12 was seen at 400 ng C mL^–1^ (Figure 4D). Cultures at I_40_ expressed an opposite trend, reaching the highest GGE measured in this study (1.10 ± 0.35) at the lowest prey density. Finally, cultures at the highest irradiance and medium prey density also exhibited a high GGE, reaching 0.99 ± 0.43.

#### Cellular Chl-*a* and Photosynthetic Rate

**FIGURE 5:**
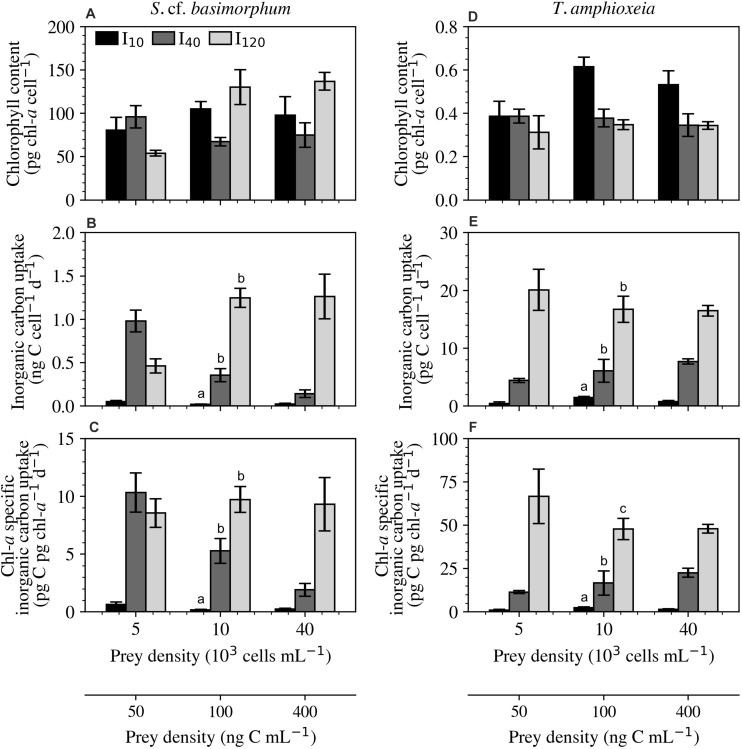
Experiment 2: Physiological responses of *Strombidium* cf. *basimorphum* **(A–C)** and *T. amphioxeia* **(D–F)** at the three light (I_10_, I_40_, and I_120_) and three prey density treatments (50, 100, and 400 ng C mL^–1^). **(A,D)** Cellular chl-*a* content. **(B,E)** Daily cellular inorganic carbon uptake rate. **(C,F)** Daily chl-*a* specific inorganic carbon uptake rate. Black, dark gray, and light gray bars denote light treatments I_10_, I_40_, and I_120_, respectively. Data points are means ± STD (*n* = 9). Prey density was not proven to be statistically significant for any of the displayed variables; the letters, therefore, represent statistically similar groupings based only on the overall effects of irradiance across all prey density treatments. Subplots without letters did not show significant effects of prey density or irradiance.

The prey species, *T. amphioxeia*, exhibited a negative correlation between cellular chl-*a* and irradiance and a positive correlation between irradiance and both cell-specific and chl-*a* specific inorganic carbon uptake rates. Cellular chl-*a* content in *S.* cf. *basimorphum* was, on average, 231 times greater than in its prey (Figures 5A,D). The large increase in chl-*a* content observed in the high-irradiance treatment at intermediate and high prey densities can be primarily explained by the substantial increase in biovolume seen in these treatments; biovolume-specific chl-*a* content at I_120_ (Supplementary Figure 2) was often actually lower than at the other two irradiances. Chl-*a* contents were similar between the two highest prey levels at any irradiance. The intermediate light level is the only one to exhibit a decrease in chl-*a* content from the low to intermediate prey densities.

In the lowest irradiance, regardless of prey density, the cell- and chl-*a* specific inorganic carbon uptake rates (Figures 5B,C) were extremely low when compared with the other two light levels. This trend is also seen in the prey species at the lowest light level (Figures 5E,F). Ciliates grown at I_40_ reached a maximum inorganic carbon uptake rate of 979 ± 125 pg C cell^–1^ d^–1^ at the lowest prey density and a minimum of 141 ± 45 pg C cell^–1^ d^–1^ at the highest prey density. Cultures kept at the highest light level expressed cellular inorganic carbon uptake rates that increased with increasing prey density to a maximum of 1260 ± 260 pg C cell^–1^ d^–1^. The chl-*a* specific inorganic carbon uptake rate of cultures kept at I_10_ and I_120_ remained relatively constant across the tested prey densities. At I_40_, however, ciliates exhibited a decreasing cell-specific and chl-*a* specific inorganic carbon uptake rate as prey availability increased. This was a stark contrast to the trends in inorganic carbon uptake rate displayed by *T. amphioxeia* at the same light level, which increased with increasing cell density.

#### Carbon Acquisition

**FIGURE 6:**
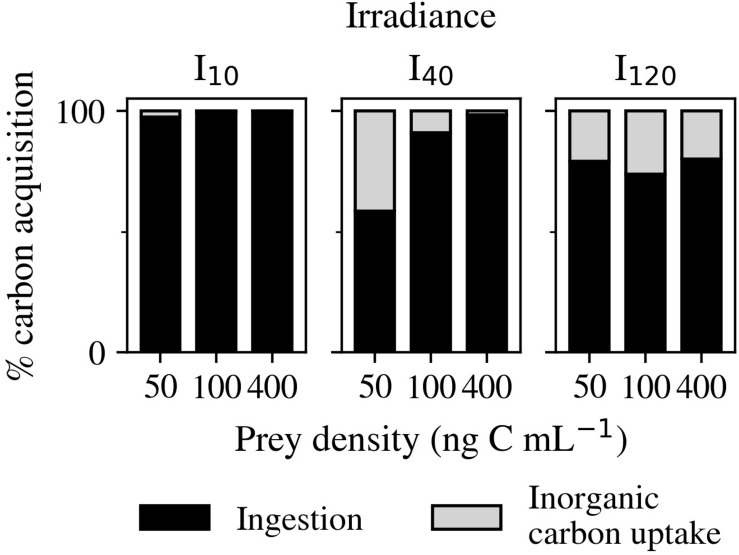
Experiment 2: Percentage of carbon acquired from inorganic carbon uptake and ingestion over the three light (I_10_, I_40_, and I_120_) and three prey density treatments (50, 100, and 400 ng C mL^–1^).

The percentage contribution of inorganic carbon uptake was found to always be lowest at I_10_ (Figure 6). At this irradiance, a minimum of 97.4% of C came from ingestion. In contrast, at I_120_, ingestion only contributed an average of 77.7% of C and experienced less variation between the different prey densities. Cultures at I_40_ had the greatest contribution from inorganic carbon uptake (41.5%) at 50 ng C mL^–1^, and the lowest (1.2%) at 400 ng C mL^–1^.

## Discussion

### Effects of Irradiance on Prey-Starved *Strombidium* spp.

The ability of *S.* cf. *basimorphum* to take up inorganic carbon (photosynthesize) led to a prolonged survival during prey scarcity when light was available. During the starvation experiment, cultures kept at I_10_ and I_40_ did not exceed 50% mortality until 4 days after complete prey depletion. Mortality rates > 90% were only reached after 11 and 13 days, respectively (Figure 2B). This is in accordance with observations made on other generalist non-constitutive mixotrophs (GNCMs) (Schoener and Mcmanus, 2012; Mcmanus et al., 2018; Maselli et al., 2020). This prolonged survival without access to prey stands in contrast to purely heterotrophic oligotrichs, which have been shown to reach almost complete culture mortality within 1–4 days (Montagnes, 1996).

While *S.* cf. *basimorphum* survived longer without prey than comparable heterotrophs, cultures still achieved nearly complete mortality within 2 weeks of prey depletion and were unable to undergo any further cell divisions at any irradiance. This is in contrast to specialist non-constitutive mixotrophic (SNCM) ciliates, such as red *Mesodinium* spp., who have been shown to not only survive for months without food but even continue to divide 3–4 times after prey is depleted (Smith and Hansen, 2007; Hansen et al., 2013). The ability of non-constitutive mixotrophs to survive for prolonged periods is likely dependent on their capacity to keep their acquired photosystems functional. To this end, many SNCMs have been shown to exhibit (among others) two important capabilities that have not yet been discovered in GNCMs: the capacity to additionally sequester prey nuclei and the ability to divide their stolen chloroplasts.

Without this higher level of plastid control, *S.* cf. *basimorphum* cultures immediately began experiencing mortality once prey was depleted. Despite this, chl-*a* specific inorganic carbon uptake rates were seen to rapidly increase directly following prey depletion and remain at approximately this peak value until experimental cessation (Figure 3C). This increase may be due to physiological starvation response, wherein individuals actively begin to photosynthesize at a higher rate when they are no longer able to ingest enough carbon from prey. A physiological response may also be suggested by the increase in the relative contribution of inorganic carbon uptake to *S.* cf. *basimorphum*’s total carbon budget at lower prey densities, as seen in this study (e.g., at I_40_). Conversely, this increase in photosynthetic ability may also derive from the ciliates digesting some of their chloroplasts when little or no prey is available, thus reducing the incidence of self-shading and potentially increasing the efficiency of any un-digested chloroplasts.

In the I_120_ treatment, the cellular chl-*a* content decreased faster than at the lower light treatments, and inorganic carbon uptake rates were approximately nine times lower than in the mid- and low-light treatments on day 5. At the high light level, the ciliate cultures reached over 80% mortality 2 days after prey depletion, bringing them closer to rates expressed by heterotrophic species. This fast decay and high turnover may indicate that *S.* cf. *basimorphum* has a decreased ability to control plastid photoadaptation and repair damages from photo-oxidative stress when compared to organisms that have innate photosystems (Lavaud et al., 2004; Funk et al., 2011). Even SNCMs have been shown to begin to deplete their chl-*a* reserves twice as quickly in high light than in low light (Johnson and Stoecker, 2005).

### Influence of Irradiance and Prey Density on *Strombidium* spp.

#### Ingestion Rate

Evidence of light-dependent ingestion rates has been reported previously among both heterotrophic and mixotrophic SNCMs dinoflagellates (Jakobsen et al., 2000; Li et al., 2000; Kim et al., 2008) and heterotrophic ciliate species (Strom, 2001; Tarangkoon and Hansen, 2011). However, our results did not indicate that ingestion rates were significantly affected by irradiance in *S.* cf. *basimorphum* and our results were comparable to what has been previously found for this species in other light conditions (Maselli et al., 2020). Similarly, Stoecker et al. (1988b) did not find light effects on ingestion in the only oligotrich ciliate studied previously, *Laboea strobila*. Nevertheless, further research is needed to confirm the presence or absence of this phenomenon in *Strombidium* spp. and other GNCM oligotrichs.

#### Inorganic Carbon Uptake Rate

It is noteworthy that inorganic carbon uptake rates decreased with increasing prey density in the I_40_ treatment (Figure 5B). Maselli et al. (2020) observed a similar pattern in *Strombidium* spp. incubated at 70 μmol photons m^–2^ s^–1^. However, inorganic carbon uptake rates actually increased as a function of prey density in the I_120_ treatment. This may indicate that the ciliates experience self-shading at lower irradiances (≤70 μmol photons m^–2^ s^–1^) when prey is abundant, as has also been suggested in a modeling study (Flynn and Hansen, 2013). These trends, in combination with the low photosynthetic rates displayed by starved cultures in higher intensity light conditions, suggest that the ciliate cannot maintain functional chloroplasts without replacement in higher intensity light conditions. This phenomenon may be a result of photoinhibition, as chloroplasts exposed to higher irradiances would work at higher rates and the ciliate may therefore require faster chloroplast turnover – via ingestion – to keep them functional. However, previous studies on other GNCM species have not shown evidence of photoinhibition in ciliates exposed to irradiances up to 500 μmol photons m^–2^ s^–1^ (Stoecker et al., 1988a; Putt, 1990a) – higher than any employed in this study.

Photosynthetic rates based on chl-*a* are known to be significantly different when comparing organisms with innate photosystems and the non-constitutive mixotrophs that utilize stolen photosystems (Stoecker et al., 1988b; Mcmanus et al., 2012; Maselli et al., 2020). When directly comparing the rate of chl-*a* specific inorganic carbon uptake in GNCMs and their prey, *S. rassoulzadegani* was shown to operate its chloroplasts at ∼52% efficiency (Mcmanus et al., 2012). In the present study, however, *S.* cf. *basimorphum* was only able to exceed 40% efficiency when subject to the lowest prey availability and the lowest two light conditions (I_10_ and I_40_). This may hint again to both the phenomena of self-shading that can occur at lower irradiances when cells are large and well-fed, as well as the ciliate’s inability to efficiently utilize its stolen chloroplasts when subject to higher irradiances.

The rates of inorganic carbon incorporation measured in this study were comparable to what has been previously found in four other *Strombidium* spp. (Stoecker et al., 1988a), but nearly eight times lower than what was measured in *L. strobila* when grown in similar conditions (Stoecker et al., 1988b). We did not study inorganic carbon uptake at irradiances >140 μmol photons m^–2^ s^–1^, but in the cases of *L. strobila* and the four other *Strombidium* species, inorganic carbon uptake rates have been shown to increase as a function of irradiance up to 1,200 μmol photons m^–2^ s^–1^ (Stoecker et al.,1988a,b).

#### Growth, Biovolume, and GGE

The growth rates of *S.* cf. *basimorphum* were positively correlated to prey density at all irradiances. This increase in growth rates was especially apparent when biovolume was accounted for (see Supplementary Figure 2A). Many heterotrophic and mixotrophic plankton have been shown to follow a similar pattern (Montagnes, 1996; Hansen et al., 1997; Smith and Hansen, 2007). GNCM ciliates seem to allocate the carbon acquired from photosynthesis into polysaccharides, which are then preferentially used for respiration (Putt, 1990b; Stoecker and Michaels, 1991). Thus, inorganic carbon uptake can help to sustain ciliate growth by covering part of the respiratory demand. This is of particular relevance when prey availability is low. In the present study, growth rates were generally lowest in the lowest light treatment (I_10_), when, in fact, cell-specific inorganic carbon uptake rates were significantly depressed. In the I_10_ treatment, indeed, *S.* cf. *basimorphum* appeared to primarily function as a heterotroph. At I_120_ when food was at or above 100 ng C mL^–1^, ciliate cells were significantly larger than at other light levels. Therefore, the larger GGE (discussed below) exhibited at higher light levels is due to an increase in biovolume and not an increase in cellular divisions. Similarly, *L. strobila* was also shown to grow to significantly greater sizes in the light, when compared to in the dark (Stoecker et al., 1988b). The highest GGE of *S.* cf. *basimorphum* at each level of prey density corresponded with the light level where inorganic carbon uptake was also greatest. When less prey was available (50 ng C mL^–1^), GGE was highest at I_40_. However, at 100 and 400 ng C mL^–1^, both photosynthesis and GGE instead saw their prey density-specific maximums at I_120_ (Figure 4D). Interestingly, other studies have reported trends in GGE similar to what was shown at I_40_. Three species of *Strombidium* (including *S.* cf. *basimorphum*) were shown to have increased reliance on inorganic carbon uptake at lower prey densities when grown at 70 μmol photons m^–2^ s^–1^ (Maselli et al., 2020) and 100 μmol photons m^–2^ s^–1^ (Schoener and Mcmanus, 2012, 2017). However, there is little record of GNCMs having a similarly dramatic increase in GGE at high light levels when prey is plentiful, as was shown in this study.

### Ecological Implications

In the field, *S.* cf. *basimorphum*, along with other GNCMs, will be subject to competition from other organisms that employ different nutritional strategies. It is likely, however, that GNCMs outperform their heterotrophic counterparts in environments where their GGE is greater than that exhibited by heterotrophs, which generally have a GGE of 0.3–0.4 (Straile, 1997). According to our experiments, this occurs consistently in both prey-saturated high-irradiance and prey-limiting intermediate-irradiance conditions. GNCMs can also capitalize on their prolonged ability to subsist through conditions with low prey availability when pure heterotrophs are unable to survive. Conversely, GNCMs will almost always have a lower GGE compared to SNCMs, which rely more heavily on photosynthesis (Hansen et al., 2013). However, GNCMs would theoretically gain an advantage over SNCMs in situations where the SNCM’s specific prey associate was unavailable or where inorganic nutrients or light were limited, thus restricting phototrophic growth (Stoecker et al., 1988b; Tarangkoon and Hansen, 2011; Hansen et al., 2013).

Our study also indicates that GNCMs like *S.* cf. *basimorphum* can grow in low-light conditions when prey concentrations are high enough to support maintenance. Other studies have corroborated these findings in nature, where GNCMs have been shown to maintain biomasses at the dark deep chlorophyll maximum that are comparable to those found in bright surface waters. Even at depths below the euphotic zone, GNCM ciliates can thrive due to their ability to function as completely heterotrophic organisms. In fact, at and below the depth of the chlorophyll maxima, where light is so low that it may strongly limit photosynthesis, GNCM organisms exhibit patterns similar to their heterotrophic counterparts, and experience less seasonal variability than in well-lit surface waters (Levinsen et al., 2000).

In temperate surface waters, from which our isolate of *Strombidium basimorphum* was collected, SNCMs and phytoplankton utilize the winter build-up of dissolved nutrients to form large spring blooms, reaching abundances nearly 20 times greater than recorded at more southern latitudes (Leles et al., 2017). Then, after phytoplankton biomass increases, GNCMs, exploiting this new prey source along with the available light and dissolved nutrients, begin to grow with high efficiency to from smaller-magnitude blooms in late spring, and achieving the greatest relative contribution to overall ciliate biomass (85–90%) in the summertime (Levinsen et al., 2000; Haraguchi et al., 2018). The ability of GNCMs to outcompete both heterotrophs and SNCMs in the summer can be linked to the flexible nutrition and high growth efficiency that is demonstrated in this study; GNCMs require less prey than heterotrophs for maximal growth and survival if exposed to adequate light and can withstand nutrient-depleted waters better than SNCMs by simply consuming more prey (Stoecker and Lavrentyev, 2018).

## Conclusion

In this study, the generalist non-constitutive mixotroph (GNCM) *S.* cf. *basimorphum* appeared to be most well-adapted to the mid-light condition (40 μmol photons m^–2^ s^–1^) when subjected to prey-starved or limited conditions. However, at slightly higher prey densities, *S.* cf. *basimorphum* exhibited the highest inorganic carbon uptake rates and GGE when grown at a higher irradiance. These results indicate that the GNCM is unable to maintain functioning prey chloroplasts without replacement in higher-intensity light conditions. Furthermore, while ingestion rates, growth parameters, and chl-*a* content were not shown to vary significantly with irradiance, light consistently influenced both cell-specific and chl-*a* specific inorganic carbon uptake rates. Our findings suggest that *S.* cf. *basimorphum* – and indeed, GNCMs in general – have a competitive advantage over specialist non-constitutive mixotrophs in prey-replete conditions, where they can rely more heavily on prey ingestion for growth. Conversely, they can out-compete their heterotrophic counterparts in prey-starved conditions where inorganic carbon uptake can be used to prolong survival or increase GGE.

## Data Availability Statement

The datasets presented in this study can be found in online repositories. The names of the repository/repositories and accession number(s) can be found below: Mendeley Data http://dx.doi.org/10.17632/wmm2wkcwwz.3.

## Author Contributions

EH was primarily responsible for drafting the manuscript and data visualization. HS performed the statistical analyses. EH and MM acquired all experimental data. MM and PJH contributed to experimental design and analysis/discussion of resulting data. All authors contributed and commented on earlier versions of the manuscript and approved the final manuscript.

## Conflict of Interest

The authors declare that the research was conducted in the absence of any commercial or financial relationships that could be construed as a potential conflict of interest.
