# Supplementary material for: Metabolic Reliance on Photosynthesis Depends on Both Irradiance and Prey Availability in the Mixotrophic Ciliate, Strombidium cf. basimorphum
Source: Front Microbiol. 2021 Jun 17;12:642600. doi: 10.3389/fmicb.2021.642600 (PMC8245785; doi:10.3389/fmicb.2021.642600)
Supplement: Supplementary file 1 [file Presentation_1.pdf]

Supplementary Material

1 Preliminary Experiment 1

1.1 Materials and Methods

This preliminary experiment was carried out to give a rough estimate of how *Strombidium* cf. *basimorphum* may react to different light treatments and to inform the choice of which prey densities to use in Experiment 2. Prey density was not adjusted during the experiment, and thus results could be skewed by early prey depletion in experimental replicates that began at lower prey densities.

Six prey densities (0.5, 1, 2, 5, 10, and 15 $\times 10^3$ cells mL^{-1}) were tested in three different light conditions (10, 40, and 120 μmol photons m^{-2} s^{-1}, referred to as I_{10}, I_{40}, and I_{120}, respectively). A sample of 20 mL of each experimental prey density was prepared by mixing concentrated algal cultures with filtered seawater. Then, each prepared algal culture was distributed into eight wells, which each contained 2 mL of the culture. The first four wells were assigned to be experimental mixed cultures, and 10 twice-washed ciliate cells were added to each of the four wells. The remaining four wells of the same prey density were designated as control *T. amphioxeia* monocultures. The experiment was initiated with the introduction and mixing of ciliates and prey in the plates as described and ended after three days when all cultures were fixed in Lugol's solution and counted to determine both the growth and ingestion rates of the ciliates. *T. amphioxeia* densities were determined using Sedgewick-Rafter chambers, counting a minimum of 200 cells.

The resulting response curve for growth rate was fitted to a Michaelis-Menten equation (Supplementary Equation 1), as modified by (Montagnes, 1996).

$$\mathrm{V} = \frac{V_{max} * [C - T]}{K_m + [C - T]} \qquad \textit{Sup. Eq. 1}$$

V is the ciliate's growth rate, V_{max} is their maximum possible growth rate, C is the algal/prey density, T refers to the prey density at which ciliate growth rate is 0, and K_m is the prey density at which V is exactly half of V_{max}.

The subsequent Michaelis-Menten Kinetics curves were compared between irradiances using an extra-sum-of-squares F-test. This test separately compared the pooled sum of squares from the curves for each of the light treatments to the extra sum of squares for their combined data fit to a single common curve. This was also done separately for parameters V_{max}, K_m, and T. The null hypothesis is that a single curve or parameter estimate provides a better fit for the three data sets, rather than separate curves or parameters (Motulsky and Ransnas, 1987). A p-value of 0.05 was used for significance testing. Both the curve fitting and subsequent analysis were done utilizing GraphPad Prism version 8.4.2 for Mac.

1.2 Results

As these experiments were meant to give a rough estimation of light and prey effects, prey density was not as strongly controlled as in Experiment 2. The prey density over the three days of this experiment was quite variable (see Supp. Table 1), limiting the statistical power of the subsequent findings.

Supplementary Table 1. *Prelim. Experiment 1. Initial and final prey densities at the three experimental light treatments. All numbers are in algal cells mL^{-1} unless otherwise denoted. Standard errors are displayed in parentheses.*

Initial prey density (10^3 algal cells mL^{-1})	10 (μmol photons m^{-2} s^{-1})	40 (μmol photons m^{-2} s^{-1})	120 (μmol photons m^{-2} s^{-1})
0.5	198 (12.0)	210 (46.6)	162.5 (10.5)
1	240 (7.36)	300 (45.6)	225 (19.3)
2	338 (19.0)	375 (26.3)	225 (17.6)
5	375 (17.6)	300 (31.9)	600 (73.6)
10	225 (17.6)	350 (20.4)	475 (22.8)
15	260 (14.7)	950 (119)	650 (22.5)

With that in mind, prey density was shown to have a significant impact on both growth and ingestion rates, while light did not appear to affect either variable (p-value = 0.052). Growth rate (Supp. Figure 1A) increased from 0.03 d-1 at the lowest initial prey density (5 ng C mL^{-1}) to 0.42 d^{-1} at the highest prey density (150 ng C mL^{-1}). Ingestion rates ranged from 26.9 prey cells predator^{-1} d^{-1} at the lowest initial prey density and 481 prey cells predator^{-1} d^{-1} at the highest.

The growth rate data were fitted to a Michaelis-Menten kinetics curve for each of the three light levels (Supp. Figure 1A, Supp. Table 2). The resulting curves indicated that ciliates grown at 10 μmol photons m^{-2} s^{-1} required the highest prey threshold density (T) to sustain the population. Cultures grown at the highest light treatment, 120 μmol photons m^{-2} s^{-1}, required the lowest density of prey to achieve half their maximum growth (*K_m*).

***Supplementary Table 2**. Prelim. Experiment 1. Mean values for the three parameters of the modified Michaelis-Menten equation fit to data on growth vs. food concentration for each experimental light treatment. Standard errors are denoted inside parentheses.*

Light treatments (μmol photons m^{-2} s^{-1})	10	40	120	p-value
Overall Fit	-	-	-	<0.0001
K_m	7.99 (4.63)	2.73 (1.18)	2.32 (0.96)	0.118
V_{max}	1.67 (0.71)*	0.59 (0.12)*	0.69 (0.12)*	0.015
T	0.35 (0.07)	0.30 (0.11)	0.18 (0.095)	0.307

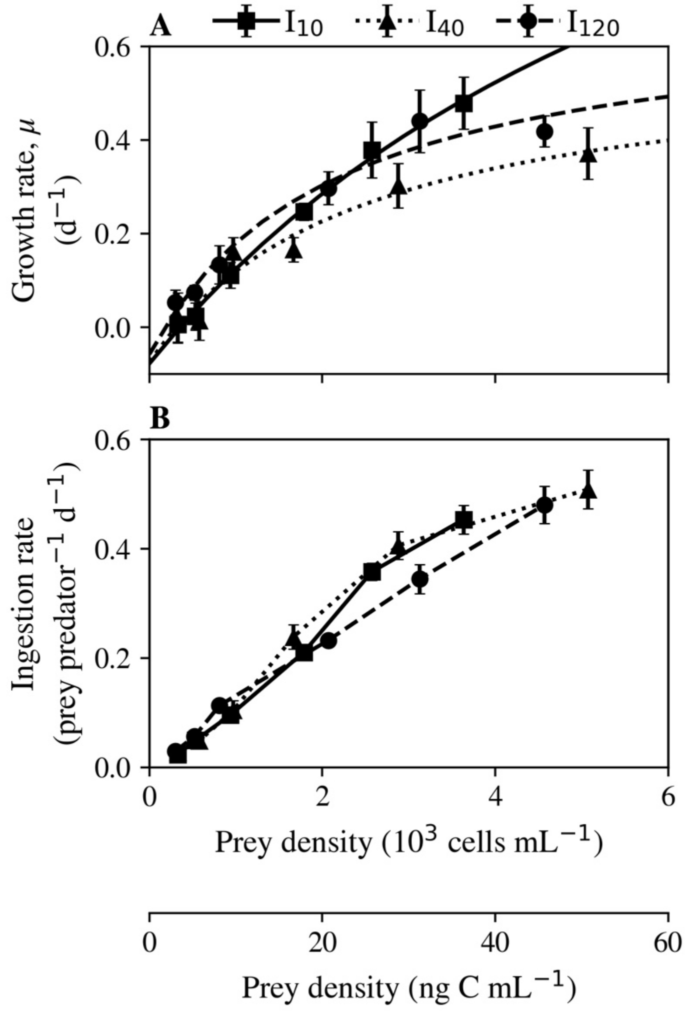

Supplementary Figure 1. Prelim. Experiment: Growth rate$_{\mu}$ (A) and ingestion (B) responses of *S.* cf. *basimorphum* across three different light treatments and six different prey densities. Growth rate response curves were numerically fitted to Michaelis-Menten kinetics, and curves in (A) represent the subsequent models. Curves in (B) connect average ingestion rates at each prey density to each other. Solid, dashed, and dotted lines denote light treatments I_{10}, I_{40}, and I_{120}, respectively.

2 Experiment 2: Data controlled for biovolume

Upon finding large variability in the size of ciliates acclimated to different light and prey conditions (Supplementary Figure 2; Supplementary Table 3), it was clear that simply reporting cell-specific results for variables such as growth rate and chl-*a* content would not accurately capture the full implications of *S.* cf. *basimorphum*'s physiologic changes (Supplementary Figure 2). Therefore, to determine the amount of additional volume gained by ciliate cells (in $\mu m^3\ d^{-1}$) the growth rate (in cellular divisions d^{-1}) for each culture was multiplied by the average cell biovolume (Supplementary Figure 3A). Similarly, to compare chl-*a* content across cells of different sizes, volume-specific chl-*a* (in pg chl-*a* $10^{-3}\ \mu m^{-3}$) was calculated by dividing the cell-specific chl-*a* content by the average cell biovolume (Supplementary Figure 3B).

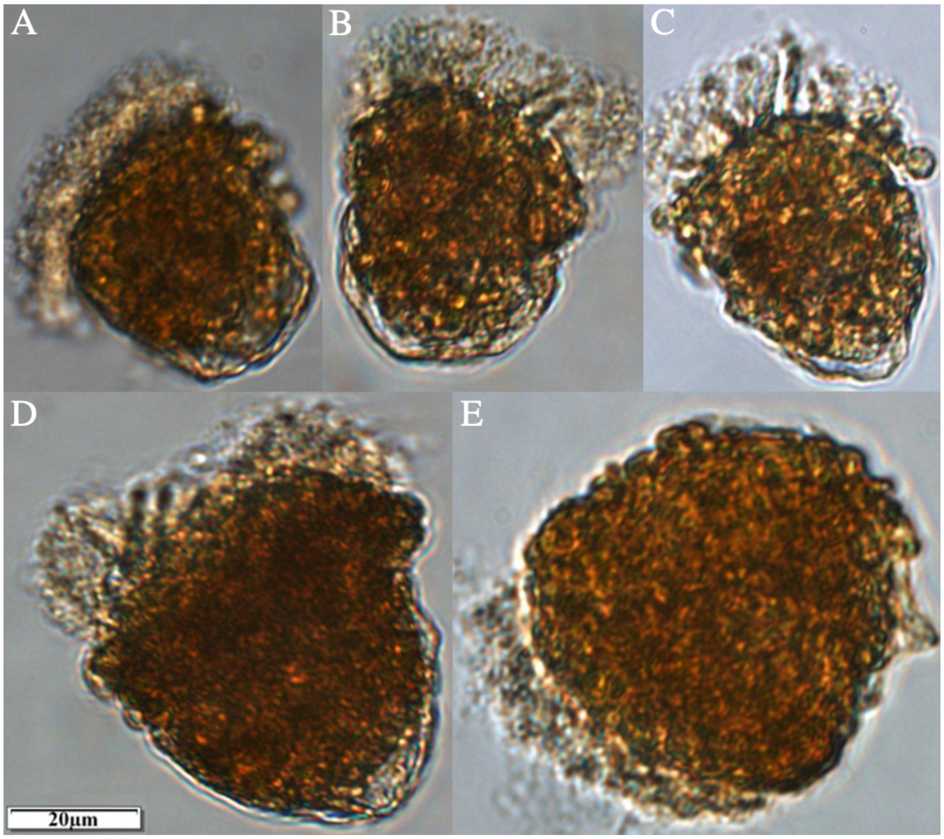

Supplementary Figure 2. Images of different *S.* cf. *basimorphum* cells fixed with Lugol solution. The top row of images (A, B, and C) shows examples of small-sized cells that were grown in prey starved or limited conditions. The two bottom pictures (D and E) show cells that were grown in prey replete conditions.

Supplementary Table 3*. Experiment 2. Average cell sizes for each of the nine experimental treatment conditions. Standard errors are denoted in parentheses (n = 90).*

Light Condition (μmol photons $m^{-2} s^{-1}$)	Prey Condition (10^3 cells mL^{-1})	Length (μm)	Width (μm)	Biovolume ($10^3 \mu m^{-3}$)
	5	37.42 (0.518)	34.59 (0.418)	24.16 (0.818)
10	10	37.42 (0.652)	35.62 (0.894)	27.79 (1.672)
	40	39.55 (0.549)	37.55 (0.459)	30.26 (1.162)
	5	38.04 (0.494)	39.78 (0.433)	32.28 (0.931)
40	10	35.72 (0.419)	35.12 (0.378)	23.59 (0.734)
	40	40.63 (0.526)	39.60 (0.417)	34.18 (1.062)
	5	37.58 (0.404)	37.15 (0.482)	27.81 (0.828)
120	10	45.04 (0.512)	45.02 (0.482)	48.97 (1.503)
	40	44.48 (0.584)	45.28 (0.549)	50.00 (1.537)

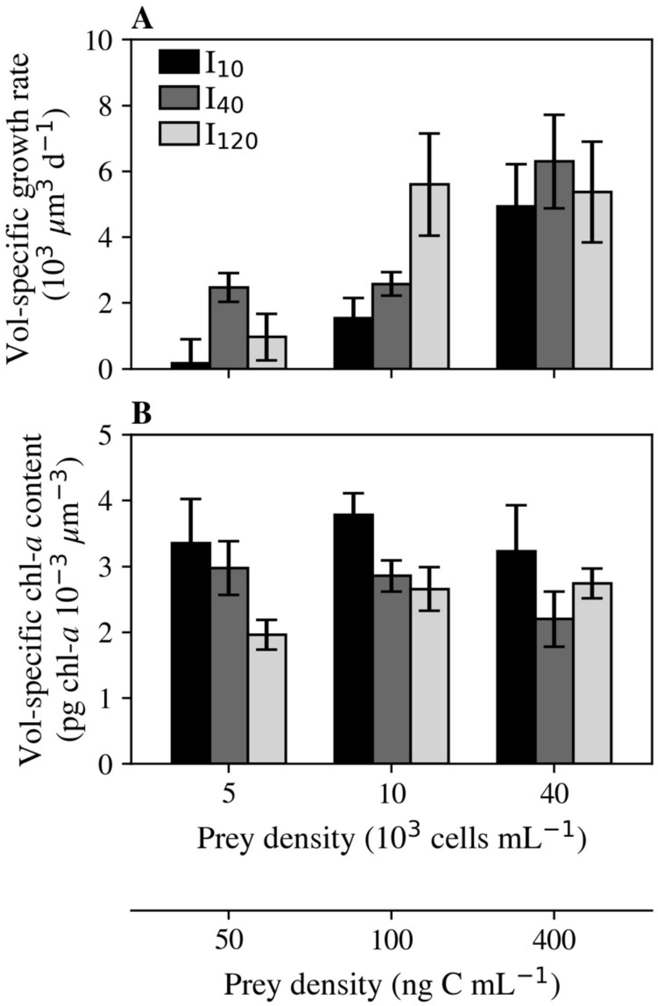

Supplementary Figure 3. Experiment 2: (A) Biovolume-specific growth rate. (B) Biovolume-specific chl-*a* content across the three experimental light levels and prey densities. Black, dark grey, and light grey bars denote light treatments I_{10}, I_{40}, and I_{120}, respectively.

References

Montagnes, D. J. S. (1996). Growth responses of planktonic ciliates in the genera *Strobilidium* and *Strombidium*. *Mar. Ecol. Prog. Ser.* 130, 241–254.

Motulsky, H. J., and Ransnas, L. A. (1987). Fitting curves to data using nonlinear regression: a practical and nonmathematical review. *FASEB J.* 1, 365–374. doi:10.1096/fasebj.1.5.3315805.
